# The role of soluble thrombomodulin (sTM) in risk stratification of hemorrhagic fever with renal syndrome and prognostic assessment

**DOI:** 10.1371/journal.pntd.0014132

**Published:** 2026-03-20

**Authors:** Min Wei, Zhuoran Xiao, Jiaojiao Cao, Xinyi Du, Mengyuan Li, Rongrong Zhang, Xiaofei Yang, Shasha Wu, Chao Fan, Jing Zhang, Jianqi Lian, Chuantao Ye

**Affiliations:** 1 Department of Infectious Diseases, The Second Affiliated Hospital of Air Force Medical University, Xi’an, Shaanxi, China; 2 Department of Traditional Chinese Medicine, The First Affiliated Hospital of Air Force Medical University, Xi’an, Shaanxi, China; Washington State University, UNITED STATES OF AMERICA

## Abstract

**Objective:**

The increase in vascular permeability and bleeding caused by systemic endothelial damage is the most basic pathological change of hemorrhagic fever with renal syndrome(HFRS) caused by Hantavirus, but there is a lack of early biomarkers to detect the severity and prognosis of HFRS. Soluble thrombomodulin (sTM) is a sensitive marker of endothelial damage. In this study we investigated the role of sTM in the evaluation of the severity and prognosis of HFRS patients.

**Methods:**

A retrospective analysis was conducted on 51 patients with HFRS treated in the Infectious Diseases Department of the Second Affiliated Hospital of Air Force Medical University from January 2023 to January 2024. We compared the changes of sTM among patients with HFRS in different phases and subtypes, and analyzed the correlation between sTM and related laboratory diagnostic indicators. Additionally, we evaluated the differences of sTM and related laboratory diagnostic indicators between survivors and non-survivors, and assessed the predictive ability of sTM for the mortality risk of HFRS patients based on the ROC curve, and determined the cut-off value of sTM.

**Results:**

In the HFRS phases, sTM increased during the febrile phase, reached its peak during the hypotensive phase, and decreased during the oliguria-dipuria phase. In the HFRS subtypes, there was no difference between the mild and moderate subtypes (*P* > 0.05), while in the moderate, severe, and critical subtypes, it showed a “stepwise” increase (*P* < 0.05), with the greatest jump being from “severe” to “critical” (*P* < 0.0001). Linear regression analysis revealed a strong positive correlation between sTM and SOFA scores (R² = 0.6076, P < 0.0001). The level of sTM in the non-survivor group (62.25 (54.60, 81.98) TU/mL) was significantly higher than that in the survivor group (28.10 (21.40, 44.15) TU/mL). The ROC curve indicated that the AUC of sTM was 0.9317, which had a high predictive value for the mortality risk of HFRS patients and was second only to SOFA (0.9309), and the combined AUC could reach 0.9492. When the cut-off of sTM was 49.98 TU/mL, the sensitivity and specificity were 81.36% and 100%. In addition, the Kaplan-Meiers survival analysis showed that when sTM ≥ 49.8 TU/mL, the 28-day mortality rate of HFRS patients was higher (*P* < 0.01).

**Conclusion:**

sTM serves as a valuable biomarker for risk stratification and prognosis evaluation in HFRS.

## Author summary

Hantavirus is the causative agent of hemorrhagic fever with renal syndrome (HFRS), a disease characterized by the increased permeability and bleeding of tiny blood vessels. Early detection of this vascular damage is challenging, yet it plays a decisive role in determining which patients will progress to a critical condition. We investigated whether a natural blood protein, sTM- a biomarker that endothelial cells release as a silent alarm upon injury-could serve as an early warning indicator for HFRS. In a study involving 51 HFRS patients, we observed that sTM levels increase in a stepwise manner in accordance with the severity of the disease. Specifically, the more severe the symptoms, such as shock and kidney failure, the higher the sTM levels. Notably, sTM levels in patients who ultimately succumbed to the disease were twice as high as those in survivors. A simple blood test measuring sTM upon patient admission was able to identify severe cases with 91% accuracy. A normalized sTM value above 49.80 was the most predictive of a poor prognosis. Given that sTM is a stable biomarker and can be easily quantified using routine hospital analyzers, it has the potential to be immediately implemented for the triage of HFRS patients.

## Introduction

Hemorrhagic fever with renal syndrome (HFRS) is a natural zoonotic disease caused by viruses belonging to the Hantavirus family and the Orthohantavirus genus. Rodents are the main source of infection for this disease. Its main manifestations include fever, capillary leakage, hemorrhage, hypotension, and renal impairment [1,2]. HFRS occurs worldwide, but China is the most heavily affected country, accounting for more than 90% of global cases. Two hantavirus types circulate in China: Hantaan virus (also called type I or “field-rodent type”), carried mainly by the apodemus agrarius, which causes severe disease; and Seoul virus (also called type II or “house-rodent type”), carried primarily by the brown rat, which produces milder illness. Shaanxi Province is a mixed epidemic area in which Hantaan virus overwhelmingly predominates. HFRS occurs throughout the year, with two seasonal peaks in spring and autumn-winter. The disease predominantly affects young and middle-aged men, with a male-to-female ratio of approximately 3:1 [3]. HFRS is a severe systemic inflammatory disorder, endothelial injury increases vascular permeability, resulting in plasma leakage, secondary edema, hypovolemic shock, acute kidney injury, coagulopathy, and even multiple organ dysfunction syndrome. Despite the implementation of early fluid resuscitation and vasoactive drugs, the mortality rate among HFRS patients remains elevated, particularly in those who are critically ill [4,5]. Therefore, timely identification of individuals at risk for developing more severe or critical illness is essential for enhancing clinical outcomes [6–8]. In this context, the utilization of biomarkers is suggested to enhance clinicians’ capacity to stratify the risk of HFRS patients.

Endothelial dysfunction plays a crucial role in the pathogenesis of HFRS [9]. The increased vascular permeability results in plasma exosmosis, platelet consumption, and coagulation disorders, which are associated with various clinical features in HFRS, including conjunctival congestion and edema, pulmonary edema, soft palate and scattered bleeding points on the skin, hypotension shock, and oliguria [3,4,10]. Thrombomodulin (TM), a transmembrane glycoprotein expressed abundantly on the endothelial cells, is released from the endothelial surface when it is damaged leading to a significant increase in soluble thrombomodulin (sTM) concentrations [1]. Therefore, sTM is considered to be a sensitive marker of endothelial damage [11,12].Several previous studies have demonstrated the association between endothelial dysfunction markers and sTM. Khan et al. conducted a case control study involving 84 seriously ill septic surgical patients and revealed significantly higher levels of sTM in seriously ill surgical septic patients, which positively correlated with endothelial dysfunction and organ damage [13]. Similarly, Chen et al. showed that the levels of sTM were significantly increased in patients with severe acute pancreatitis, which were closely related to microcirculation disorders and poor prognosis [14].

The primary objectives of this study were to examine the utility of sTM in assessing the severity and predicting the prognosis of HFRS patients.

## Materials and methods

### Ethics statement

Ethical approval for this research was granted by Ethics Committee of Second Affiliated Hospital of Air Force Medical University (No.201912-07). and the written informed consents were obtained from the participants or their guardians.

### Patients

This retrospective cohort study evaluated patients admitted to the Second Affiliated Hospital of Air Force Medical University (Xi’an, China) from January 2023 to January 2024. All the enrolled patients met the following inclusion criteria: (1) Consistent with the typical clinical manifestations of HFRS and confirmed by serological examination; (2) 18–80 years old; (3) In the acute phase at the time of hospital admission. In addition, the patients according with any of the following criteria were excluded: (1) Age > 80 or < 18 years; (2) Complicated with pregnancy, chronic kidney diseases, autoimmune diseases, bacterial infection, viral hepatitis or other infectious diseases.

### Data collection and Availability

In this study, all clinical and laboratory data were first collected within 24 hours after the patient’s admission to the hospital. Subsequently, they were collected again as soon as possible within 24 hours after the patient entered a certain phase of HFRS (fever, hypotension, oliguria, polyuria), including sTM, white blood cells (WBC), neutrophils (NEU), red blood cells (RBC), lymphocytes (LYM), platelets (PLT), procalcitonin (PCT), interleukin-6 (IL-6), C-reactive protein (CRP), activated partial thromboplastin time (APTT), total bilirubin (TBIL), and serum creatinine (Scr). The original data was submitted as an attachment (S1 Data).

### Clinical classification of HFRS [3,15,16]

The specific clinical phsaes criteria were showed in Table 1. Furthermore, based on the severity of the symptoms of HFRS, we also categorized the patients included in the study into the following four types: (1) Mild-type: Patients with slight symptoms and mild renal impairment (proteinuria or hematuria ranging from + to ++), and without obvious oliguria and hypotension.(2) Mmoderate-type: Patients with typical symptoms of effusion (bulbar conjunctiva) and petechiae (skin and mucous membranes), and also meeting with obvious oliguria and kidney injury (proteinuria or hematuria more than +++), some of them presenting with transient hypotension.(3) Severe-type: Patients presenting with the following symptoms: A. severe effusion (bulbar conjunctiva and either pleura orperitoneum) and haemorrhage (skin and mucous membranes); B. significant hypotension and uremia; C. AKI with oliguria (urine output 100 ~ 500 mL/day) ≤5 days or anuria (urine output<100 mL/day)≤2 days; (4) On the basis of the severe-type, patients meeting with any of the following complications: refractory shock (≥2days), visceral hemorrhage, heart failure, pulmonary edema, brain edema, severe secondary infection, and severe AKI with either oliguria (urine output 100 ~ 500 mL/day) > 5 days or anuria (urine output <100 mL/day) > 2 days.

**Table 1:** Clinical phases of HFRS.

	Febrile	Hypotensive	Oliguric	Polyuric	Convalescent
**Onset time**	2nd ~ 3rd day	3rd- 7th day	5th-8th day	9th-14th day	3rd-4th week
**Duration**	4 to 6 days	Ranging from several hours to several days	2 to 5 days	1-2 weeks	1-3months
**Symptoms**	Fever, headache, back pain, orbital pain, generalized muscle and joint pain, nausea, vomiting, abdominal pain.	Hypotension, palpitations, shortness of breath, dizziness, weakness, cold limbs, fast pulse, consciousness disorders	Oliguria or anuria, hypervolemic syndrome, azotemia, metabolic acidosis,and electrolyte imbalance	Increased urine output, dehydration, electrolyte imbalance, and even secondary shock	Fatigue
**Physical** **examination**	The conjunctiva of the eyes, the skin of the face, the neck and the anterior chest are congested and flushed. There are bleeding points on the mucosa of the soft palate and the posterior wall of the pharynx. There is often effusion in the serous cavities.	Signs of exudation and bleeding tendency are obvious	Signs of exudation and bleeding tendency are obvious	Signs of exudation and bleeding tendency are decrease	Negative

### Calculation of the Sequential Organ Failure Assessment (SOFA) score

The SOFA score is composed of 0–4 points allocated to each of six organ systems based on the ratio of PaO_2_ to fraction of inspired oxygen, Glasgow Coma Scale score, mean arterial pressure, serum creatinine level, bilirubin level, and platelet count. The SOFA score ranges from 0 to 24 points, with higher scores indicating poorer organ function. The variables utilized for calculating the SOFA score and characterizing the patients were obtained through a review of electronic medical records.

### Detection of sTM levels in serum

Collect plasma using 3.8% sodium citrate (sodium citrate:blood = 1: 9) as an anticoagulant was centrifuged for 15 minutes at 1,000 × g at 4 °C within 30 minutes of collection. 50 μL of plasma sample was incubated with 50 μL of paramagnetic microparticles (Cloud-clone, Wuhan, China) coated with monoclonal anti-Thrombomodulin antibodies for 10 min at 37 °C under continuous gentle mixing. After magnetic separation and three washes with phosphate-buffered saline (PBS, pH 7.4) containing 0.05% Tween-20, 50 μL of Acridinium Ester(AE)-labeled detection antibody was added and incubated for an additional 10 min at 37 °C. Following a second wash cycle, 100 μL of chemiluminescent substrate was injected into each reaction vessel and the chemiluminescence signal was measured in a microplate luminometer.

### Outcomes of the study

The primary endpoint of the study was the 28-day all-cause mortality. Hospital records for all participants were monitored for a period of 28 days post-enrollment or until death. In cases where patients were discharged from the hospital prior to the completion of the 28-day period, follow-up was conducted through telephone interviews.

### Statistical analysis

Non-normally distributed data were presented as the median (25–75th percentiles). The Kruskal-Wallis test was utilized for multi-group comparisons, and two-group comparisons were conducted with the Mann-Whitney U test. Linear regression analysis was used to evaluate the relationship between sTM and laboratory diagnostic indicators. Prognostic prediction utilizing sTM and laboratory diagnostic indicators was assessed and compared using the ROC curve and corresponding AUROC. overall survival were calculated by the Kaplan–Meier Method. Statistical significance was determined at a level of *P* < 0.05 for all results.

## Results

### General situation of the study object

From January 2023 to January 2024, a total of 51 patients diagnosed with HFRS were included in the study. According to the typical clinical manifestations observed during the disease course, we classified HFRS into 5 phases: Febrile, Hypotensive, Oliguric, Polyuric and convalescent. The vast majority of patients experienced all 5 phases, whereas only a few—especially those who received early and prompt treatment—skipped one or more phases. In this study, it is considered that once patients enter the convalescent phase of HFRS, their mental status, appetite, and physical strength gradually improve, and renal function progressively normalizes. Specifically, daily urine volume declines to <2000 ml, while BUN and serum creatinine approach the normal range. Because these individuals are typically discharged or transferred to outpatient follow-up within a very short in-hospital window, the sampling opportunity is narrow and the rate of missing data is high. Forcibly including them would introduce substantial selection bias and compromise the internal validity of the study. Consequently, we did not enroll patients in the convalescent phase. Fianlly, we longitudinally collected sTM levels throughout the first four phases of HFRS: Febrile (n = 23), Hypotensive (n = 28), Oliguric (n = 41), and Polyuric (n = 36). Furthermore, according to the overall disease severity observed throughout the entire clinical course, patients were categorized as Mild (*n* = 4), Moderate (*n* = 15), Severe (*n* = 14) and Critical (*n* = 18). The baseline characteristics of the entire study population are presented in Table 2. The median age was 47.50 years with an interquartile range of 33.25-57.75 years, and male gender accounted for 84.62% of the patient cohort.

**Table 2:** General situation of the study object.

	Allpatients(n = 51) M(P25, P75)	Mild(n = 4) M(P25, P75)	Moderate(n = 15) M(P25, P75)	Severe(n = 14) M(P25, P75)	Critical(n = 18) M(P25, P75)
Gender (male/female)	43/8	3/1	12/3	14/0	14/4
Age(years)	49.00(34.00, 57.00)	43.50(31.25, 49.75)	57.00(34.00, 67.00)	41.50(29.75, 56.25)	47.50(35.00, 53.25)
CRP(mg/L)	20.61(10.97, 31.52)	22.74(7.91, 64.75)	20.74(12.48, 35.54)	7.18(14.65, 35.48)	18.92(8.60, 23.55)
IL-6(pg/mL)	29.32(11.35, 70.80)	39.05(16.04, 80.85)	25.14(11.35, 41.18)	14.37(8.83, 49.82)	60.55(22.24, 152.67)
PCT(ng/mL)	4.21(1.09, 10.44)	0.73(0.32, 0.84)	1.77(0.68, 7.17)	4.40(1.35, 10.75)	9.12(3.03, 22.27)
sTM(TU/mL)	35.60(23.80, 54.40)	13.70(10.13, 13.98)	25.00(21.40, 31.90)	40.15(32.73, 48.85)	57.65(50.78, 65.33)
WBC(×10E9/L)	15.62(9.21, 32.12)	6.58(5.50, 8.29)	11.08(8.48, 13.91)	17.15(10.87, 27.81)	32.96(22.19, 52.08)
NEU(×10E9/L)	8.62(6.03, 18.77)	3.97(2.93, 6.41)	6.25(4.41, 7.61)	8.30(7.04, 16.59)	20.88(12.98, 34.41)
LYM(×10E9/L)	5.00(2.69, 7.35)	1.82(1.23, 1.88)	3.64(1.95, 5.00)	3.89(2.74, 7.35)	7.40(5.68, 10.54)
PLT(×10E9/L)	36.00(22.00, 82.00)	101.00(51.25, 121.50)	75.00(26.00, 143.00)	36.50(23.50, 76.50)	23.00(17.00, 31.25)
RBC(×10E9/L)		4.53(3.74, 5.60)	3.83(3.07, 4.66)	4.43(3.75, 5.21)	4.20(3.04, 5.19)
APTT(sec)	48.60(35.40, 72.50)	37.55(29.60, 40.60)	36.40(27.27, 51.08)	51.25(30.53, 68.70)	73.05(52.05, 87.95)
Scr(umol/L)	287.50(172.10, 370.20)	72.55(9.82, 19.14)	252.60(172.10, 389.00)	362.60(234.30, 531.15)	298.30(203.33, 353.48)
TBIL(umol/L)	11.91(9.49, 19.67)	13.28(10.24, 21.69)	9.50(7.00, 11.87)	11.36(7.81,13.99)	19.55(10.95, 25.81)
SOFA(score)	8.00(6.00, 12.00)	3.00(2.00, 4.00)	6.00(5.00, 8.00)	9.00(7.75, 12.00)	12.50(7.75, 16.00)

### sTM in HFRS patients

**Fig 1:**
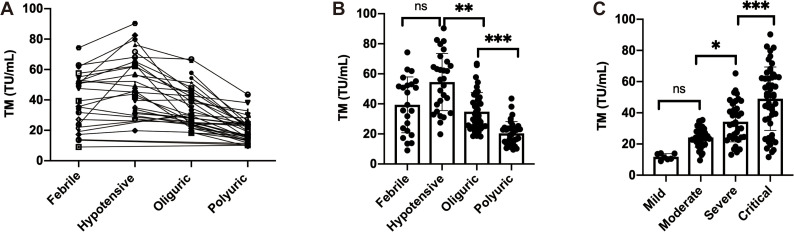
The levels of sTM in the plasma of patients with HFRS in different classifications. (A)The dynamic changes in the level of sTM in the plasma of HFRS patients. (B) sTM in distinct phases of HFRS (febrile, hypotensive, oliguric phase and polyuria). (B) sTM in HFRS patients categorized by disease severity (mild, moderate, severe, critical).

HFRS is an acute infectious disease characterized by endothelial cell dysfunction, which leads to plasma extravasation, high permeability, and sometimes even bleeding. However, studies on the levels of sTM during different classification of HFRS are relatively scarce. To comprehensively evaluate the role of sTM in the risk stratification and prognosis assessment of HFRS, we first conducted a dynamic analysis of the sTM in the plasma of HFRS patients (Fig 1A), finding that sTM increased during the fever phase and reached its peak during the hypotension phase. As the disease progressed, it gradually decreased at the oliguric and polyuria phase. However, in terms of the differences between each phase (Fig 1B), there was no significant difference between the fever phase and the hypotension phase (*P* = 0.0683 > 0.05), and compared with the hypotension phase, the sTM was lower in the oliguria phase, which was statistically significant (*P* = 0.0036 < 0.01). Moreover, compared with the oliguria phase, the plasma sTM further decreased in the polyuria phase, and both showed a high statistical significance (*P* < 0.0001). This is consistent with the mechanism of vascular endothelial injury in HFRS, that is, during the fever stage, the virus enters the blood and begins to attack the vascular endothelium, resulting in an increase in sTM release, and during the hypotension phase, the vascular endothelial damage is the most severe, with obvious microcirculation disorders, and the sTM also reaches its peak accordingly. After entering the oliguria stage, as the vascular endothelium gradually repairs, sTM gradually decreases, fully indicating that sTM can reflect the prognosis of HFRS in real time. Secondly, we also compared the changes of sTM in different subtypes of HFRS (Fig 1C), finding that there was little difference between the mild and moderate types (*P* = 0.1806 > 0.05), while in the moderate, severe, and critical types, it increased in a “stepwise” manner, with the greatest jump from “severe to critical” (*P* < 0.0001). This to some extent indicates that when sTM significantly increases, it is necessary to be highly vigilant of the rapid progression of the disease to the critical type, suggesting that sTM can be used as an early warning and precise classification laboratory tool.

### The correlation between sTM and laboratory diagnostic indicators of HFRS

**Fig 2:**
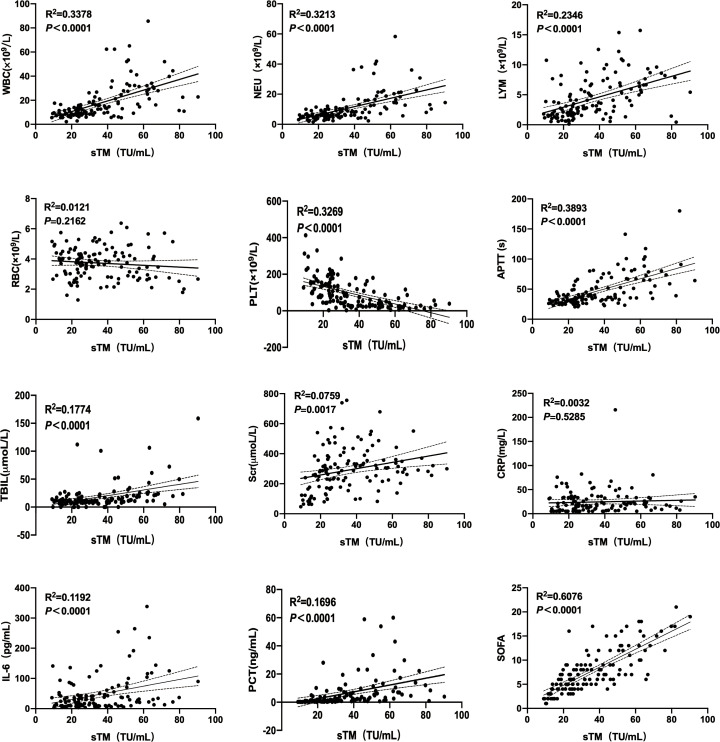
The correlation analysis between sTM and laboratory diagnostic indicators of HFRS.

In the laboratory diagnosis of HFRS, the typical “three excesses and one deficiency” can be observed in the blood routine test. Specifically, there is an increase in WBC, RBC, and LYM counts, while the PLT count decreases. Additionally, abnormalities in renal function, coagulation, and inflammatory indicators may also occur. To verify whether sTM can serve as a “summary” indicator for vascular endothelial damage in HFRS, this study further analyzed the correlation between sTM and the aforementioned laboratory indicators(Fig 2). Linear regression analysis revealed a strong positive correlation between sTM levels and SOFA scores (R^2^ = 0.6076, *P* < 0.0001), indicating that elevated sTM is associated with more severe organ dysfunction andsTM can serve as a potential biomarker for the severity of HFRS, especially when used in combination with the SOFA score. Moderate linear correlations were also observed between sTM and APTT (R^2^ = 0.3893), WBC (R^2^ = 0.3378), PLT (R^2^ = 0.3263), and NEU (R^2^ = 0.3213) (all *P* < 0.0001), suggesting that endothelial injury is closely linked to coagulation abnormalities and inflammatory responses. Weak but significant associations were found with TBIL, PCT, and IL-6, whereas no significant linear relationships were detected with RBC or CRP (*P* > 0.05).

### Comparison between survival and non-survival group

**Fig 3:**
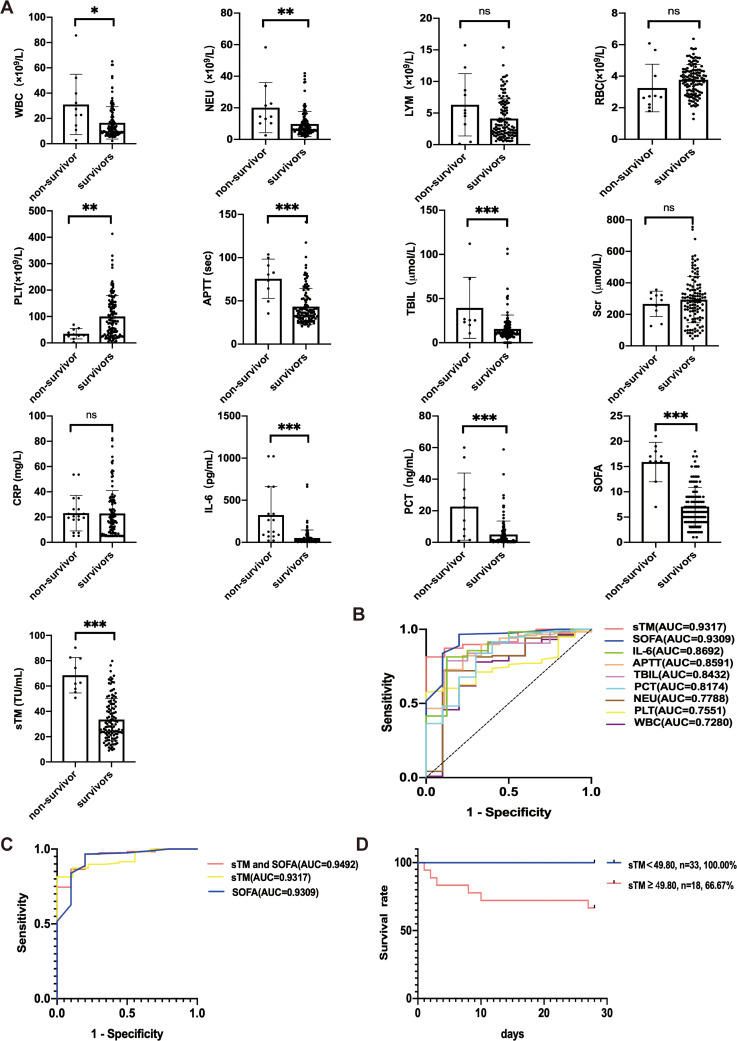
Prognostic value of sTM in HFRS. **(A)** Comparison of sTM and laboratory diagnostic indicators between survivor and non-survivor group. **(B)** Receiver operator curves generated for patients with HFRS for sTM and laboratory diagnostic indicators. **(C)** Receiver operator curves generated for patients with HFRS for the combination of sTM and SOFA score. **(D)** Kaplan-Meier survival curves for patients with the best cut-off value of sTM.

All patients diagnosed with HFRS were further categorized into survivors (n = 45) and non-survivors (n = 6), and the laboratory indicators of HFRS as well as sTM levels were compared between the two groups (Fig 3A). There was no statistically significant difference in the levels of LYM, RBC, Scr and CRP between the survivors and non-survivors. However, non-survivors exhibited significantly higher sTM levels (62.25(54.60, 81.98) TU/mL) upon admission compared to the survivors (28.10(21.40, 44.15) TU/mL). Similar findings were also observed for WBC, NEU, PLT, APTT, TBIL, IL-6, PCT and the SOFA.

### Receiver operating characteristic (ROC) curve analysis

We further assessed the predictive capability of sTM levels for in-hospital mortality using ROC curves (Fig 3B). Upon comparison of the ROC curves for WBC, NEU, PLT, APTT, TBIL, IL-6, PCT, SOFA and sTM, we observed that the area under the curve (AUC) of sTM (Area = 0.9317) and SOFA (Area = 0.9309) both exceeded 0.93, ranking in the first tier and being significantly higher than those of traditional inflammatory, blood cell count, and coagulation indicators. Additionally, the combination of sTM and SOFA score yielded an AUC of 0.9492, surpassing that of sTM and SOFA alone (Fig 3C).

ROC curve analysis established 49.80 TU/mL as the best cut-off value of sTM to identify 28-day hospital mortality, with a sensitivity of 81.36%% (95% confidence interval [CI], 73.38%-87.35%) and specificity of 100% (95% CI, 70.09%-100.00%). In addition, Kaplan-Meier survival curve revealed that patients with sTM level ≥49.80 TU/mL had a slightly higher 28-day hospital mortality compared to patients with sTM level <49.80TU/mL (33.33% vs 0%, *P* = 0.008) (Fig 3D).

## Discussion

The primary pathological alteration in HFRS is vascular endothelial dysfunction, leading to increased vascular permeability, thrombocytopenia, coagulopathy, and hemorrhagic manifestations [17,18]. The molecular mechanisms underlying vascular endothelial cell injury in HFRS remain poorly understood, primarily linked to the intricate interplay between hantaviruses, host immune responses, and endothelial cells [19,20]. Nevertheless, laboratory markers directly reflecting vascular endothelial cell injury are not widely utilized in the diagnosis and assessment of HFRS.

Mature human TM is a 557-amino-acid residue, type-1 transmembrane glycoprotein encoded by the intronless gene THBD. It is expressed by endothelial cells that line all vessels (arteries, veins, capillaries, and lymphatics). TM regulates coagulation, inflammation, innate immunity and cell trafficking by assisting thrombin in activating the protein C anticoagulant system. When the vascular endothelium is injured, TM is cleaved by neutrophil elastase, releasing sTM into the bloodstream and raising plasma sTM levels. Consequently, sTM serves as a biomarker of endothelial damage and has been linked to the diagnosis and prognosis of cardiovascular disease, sepsis and disseminated intravascular coagulation [21,22]. A data from the Atherosclerosis Risk in Communities (ARIC) Study indicate that sTM concentrations are positively correlated with the degree of atherosclerosis but negatively correlated with the risk of coronary heart disease [23]. This phenomenon may reflect the different biological meanings represented by sTM concentrations in different pathological states. In atherosclerosis, sTM concentrations may more likely reflect the degree of endothelial damage, while in coronary heart disease, sTM concentrations may more likely reflect the expression level of TM on endothelial cells and its anticoagulant function. These differences may be related to the complexity of study design, population differences, and biological mechanisms. Uncontrolled inflammatory response and coagulation dysfunction are central to the pathogenesis of sepsis. TM, a key regulator of both inflammation and the coagulation system, is intimately involved in the initiation and progression of sepsis. In an observational study of 179 septic patients and 125 non-septic controls, serum sTM was markedly higher in the sepsis cohort, moreover, the highest values were seen in patients who developed sepsis-associated coagulopathy or progressed to septic shock [24], indicating that sTM is a sensitive biomarker for early recognition of sepsis and for prediction of poor outcome. Septic shock, the most critical stage of sepsis, is characterized by severe circulatory, metabolic and cellular dysfunction and carries a mortality rate approaching 50%. Studies had shown that the sTM concentration is significantly higher in septic-shock patients than in those with sepsis alone, and it is also markedly elevated in non-survivors compared with survivors, the AUC for the predictive ability of DIC score, APACHE II score, creatinine, sTM and SOFA in mortality prediction were 0.723, 0.726, 0.777, 0.803 and 0.807 respectively [25].This is also highly consistent with the results of another study conducted by the researchers [26], underscoring that sTM outperforms traditional clinical indices, offering both earlier diagnosis of sepsis and superior risk stratification. HFRS and sepsis present an almost mirror-like pathological and physiological spectrum in the four core aspects of “inflammation - vascular endothelium - coagulation - organ failure”. However, there are currently no studies on whether sTM can serve as a reliable biomarker for assessing the severity of HFRS and predicting poor prognosis. Our research results revealed that, from the perspective of phases, sTM concentration were significantly higher during the Hypotensive and Oliguric phases compared to the Febrile and Polyuric phases. This is consistent with the pathological and physiological changes of vascular endothelial cells during the progression of HFRS. Additionally, from the classification perspective, compared to Mild HFRS patients, Moderate, Severe, and Critical patients all had higher sTM concentrations. Our findings showed that, when analyzed by phases, sTM increased during the fever phase and reached its peak during the hypotension phase, with no significant difference between the two (*P* > 0.05). As the disease progressed to the oliguric and polyuric phases, sTM declined steadily, and inter-phase differences were highly significant (*P* < 0.01), mirroring the pathophysiological evolution of vascular endothelial injury in HFRS. Additionally, Viewed by subtypes, sTM were similar in mild and moderate subtype (*P* > 0.05), but increased in a “step-wise” manner among moderate, severe and critical subtypes (*P* < 0.05), with the sharpest rise from severe to critical subtype (*P* < 0.001). Moreover, sTM and SOFA proved to be excellent predictors of mortality in HFRS, with AUC of 0.9317 and 0.9309 respectively. The AUC of other related factors range from 0.7280 to 0.8682. And when the serum sTM concentration and the SOFA score are combined, their predictive ability for the mortality of HFRS patients (with an AUC of 0.9492) even exceeds that of either sTM or SOFA alone. These findings suggest that as a “barometer” of vascular endothelial damage, sTM can be used to assess the severity and prognosis of HFRS. Furthermore, the combination of sTM and SOFA can shorten the “time window” for identifying severe cases of HFRS, thereby enabling early identification and intervention, and reducing the clinical mortality rate.

The TM receptor is a versatile mediator, responsible for endothelial thromboresistance and the regulation of fibrinolysis, complement, and inflammatory pathways. In clinical studies, recombinant human sTM has demonstrated efficacy in treating disseminated intravascular coagulation [27]. Furthermore, the soluble form of TM has shown promise in mitigating ischemic renal injury and thromboembolism in rodent models [28]. Therefore, further investigation into the potential therapeutic benefits of TM for HFRS is warranted.

This study is subject to several limitations. Firstly, it is important to note that this is a single-center study with a relatively small number of cases, which may limit the generalizability of our findings. Secondly, the lack of an independent validation cohort diminishes the credibility of our results. Notwithstanding these limitations, this study represents the initial investigation into the association between sTM and disease severity of HFRS. The inclusion of additional clinical cases in future research will further elucidate the significance of sTM in early warning for HFRS.

In conclusion, our study suggests that the measurement of sTM upon admission in HFRS patients is significantly associated with disease severity. This readily available biomarker has the potential to serve as a prognostic indicator for HFRS patients in the future.

## Supporting information

S1 Data The original data of enrolled HFRS patients. (XLSX)
